# Assessment of the Early Detection of Anosmia and Ageusia Symptoms in COVID-19 on Twitter: Retrospective Study

**DOI:** 10.2196/41863

**Published:** 2023-09-25

**Authors:** Carole Faviez, Manissa Talmatkadi, Pierre Foulquié, Adel Mebarki, Stéphane Schück, Anita Burgun, Xiaoyi Chen

**Affiliations:** 1 Centre de Recherche des Cordeliers, Université Paris Cité Sorbonne Université Institut National de la Santé et de la Recherche Médicale (INSERM) UMR 1138 Paris France; 2 Health Data- and Model- Driven Knowledge Acquisition (HeKA) Inria Paris Paris France; 3 Kap Code Paris France; 4 Department of Medical Informatics Hôpital Necker-Enfant Malades Assistance Publique - Hôpitaux de Paris (AP-HP) Paris France; 5 Data Science Platform, Imagine Institute Université Paris Cité Institut National de la Santé et de la Recherche Médicale (INSERM) UMR 1163 Paris France

**Keywords:** social media, COVID-19, anosmia, ageusia, infodemiology, symptom, Twitter, psychological, tweets, pandemic, rapid stage, epidemic, information, knowledge, online health, tweets, misinformation, education, online education, ehealth, qualitative

## Abstract

**Background:**

During the unprecedented COVID-19 pandemic, social media has been extensively used to amplify the spread of information and to express personal health-related experiences regarding symptoms, including anosmia and ageusia, 2 symptoms that have been reported later than other symptoms.

**Objective:**

Our objective is to investigate to what extent Twitter users reported anosmia and ageusia symptoms in their tweets and if they connected them to COVID-19, to evaluate whether these symptoms could have been identified as COVID-19 symptoms earlier using Twitter rather than the official notice.

**Methods:**

We collected French tweets posted between January 1, 2020, and March 31, 2020, containing anosmia- or ageusia-related keywords. Symptoms were detected using fuzzy matching. The analysis consisted of 3 parts. First, we compared the coverage of anosmia and ageusia symptoms in Twitter and in traditional media to determine if the association between COVID-19 and anosmia or ageusia could have been identified earlier through Twitter. Second, we conducted a manual analysis of anosmia- and ageusia-related tweets to obtain quantitative and qualitative insights regarding their nature and to assess when the first associations between COVID-19 and these symptoms were established. We randomly annotated tweets from 2 periods: the early stage and the rapid spread stage of the epidemic. For each tweet, each symptom was annotated regarding 3 modalities: symptom (yes or no), associated with COVID-19 (yes, no, or unknown), and whether it was experienced by someone (yes, no, or unknown). Third, to evaluate if there was a global increase of tweets mentioning anosmia or ageusia in early 2020, corresponding to the beginning of the COVID-19 epidemic, we compared the tweets reporting experienced anosmia or ageusia between the first periods of 2019 and 2020.

**Results:**

In total, 832 (respectively 12,544) tweets containing anosmia (respectively ageusia) related keywords were extracted over the analysis period in 2020. The comparison to traditional media showed a strong correlation without any lag, which suggests an important reactivity of Twitter but no earlier detection on Twitter. The annotation of tweets from 2020 showed that tweets correlating anosmia or ageusia with COVID-19 could be found a few days before the official announcement. However, no association could be found during the first stage of the pandemic. Information about the temporality of symptoms and the psychological impact of these symptoms could be found in the tweets. The comparison between early 2020 and early 2019 showed no difference regarding the volumes of tweets.

**Conclusions:**

Based on our analysis of French tweets, associations between COVID-19 and anosmia or ageusia by web users could have been found on Twitter just a few days before the official announcement but not during the early stage of the pandemic. Patients share qualitative information on Twitter regarding anosmia or ageusia symptoms that could be of interest for future analyses.

## Introduction

In recent years, social media, with its widespread usage and large user base, have gained significant attention as potential sources of information for public health surveillance. The systematic collection, analysis, and interpretation of health-related information from social media allow for various applications, ranging from the spread of infectious diseases (eg, HIV, SARS, and influenza), to vaccination uptake, antibiotics consumption, and alcohol consumption. The emergence of the COVID-19 pandemic has significantly accelerated research efforts in infodemiology and infoveillance using social media data. As an example, a search on PubMed using “social media” and “symptoms” as keywords revealed 579 publications from 2012 to 2019, and after the outbreak of COVID-19, the number of publications retrieved using the same keywords increased to 423 in 2020 alone, then 611 and 622 in 2021 and 2022, respectively, around 40% of which were COVID-19–related (163/423 in 2020, 288/611 in 2021, and 279/622 in 2022), reflecting the exponential growth of studies investigating the role of social media in symptom surveillance. Among social media platforms, Twitter stands out as an event-reactive tool with high posting frequency. For example, there have been attempts to use Twitter mining to monitor vaccine adverse events, showing that, for example, sore to touch, fatigue, and headache were the most common adverse effects in the United States [1], and symptoms related with appetite, allergy, injection site, and clots in the United Kingdom [2]. During the pandemic, it has been used for discussing various dimensions of the pandemic, including epidemiology, economy, as well as clinical and emotional aspects [3,4]. While social media provides an opportunity to directly communicate health information to the public, health related testimonies posted on the internet may also be used for early detection of symptoms and diseases. As an example, Lopreite et al [5] analyzed the data from Twitter to uncover early warning signals of COVID-19 outbreaks in Europe in the winter season 2019-2020, and showed that unexpected levels of pneumonia-related tweets were raised across a number of European countries in early 2020 prior to official announcement. For example, they identified an increase in the number of tweets mentioning dry cough during the weeks leading to the peak in February 2020.

In this study, we aim to assess the early detection of anosmia and ageusia symptoms associated with COVID-19 using Twitter data, contributing to the growing field of digital epidemiology and infodemiology. We focused on these 2 symptoms because their relation with COVID-19 was unknown at the beginning of the pandemic. After the initial outbreak of COVID-19, reported findings varied across countries and time. For example, the list of symptoms at the onset of illness reported by Wuhan clinicians in early 2020 [6] only includes fever, cough, and myalgia or fatigue, sputum production, headache, hemoptysis, and diarrhea. Later on, symptoms such as anosmia, dysgeusia, headache, and muscle pain have been noted, along with more reports of central and peripheral nervous system involvement [7,8]. Anosmia, that is, the loss of the sense of smell, and ageusia, that is, the loss of the sense of taste, have been associated with SARS-CoV-2 test positivity, regardless of the population, and illness duration or complexity [9]. In March 2020, national and international institutions (eg, [10]) proposed to add these symptoms to the list of screening tools for possible COVID-19 infection. They were followed by all health authorities worldwide, including the Center for Disease Control and Prevention and the World Health Organization, who added “new loss of taste or smell” to the list of COVID-19 symptoms. Anosmia, in particular, has been seen in patients ultimately testing positive for the coronavirus with no other symptoms. Moreover, the estimated prevalence varies a lot across studies. A multicenter European study looking at patients with mild to moderate COVID-19 disease found a prevalence of 85.6% and 88% of olfactory and gustatory dysfunctions, respectively [11]. Contrasting with this high prevalence in European COVID-19 cohorts, a prevalence of only 5.1%-5.6% impairment of smell or taste (3% in severe, 6%-7% in nonsevere) was observed in China during January and February 2020 [8]. In between, a cross-sectional survey in Italy found that 33.9% of the patients admitted before March 2020 had olfactory or taste disorders [12]. Finally, a cohort study including patients with clinically diagnosed or laboratory-confirmed COVID-19 from 28 centers, representing 13 countries and 4 continents concluded that anosmia or ageusia was the second mostly self-reported neurological symptom after headache by patients with COVID-19, which led to a global prevalence of 26% [13]. This study was performed by the Global Consortium Study of Neurological Dysfunction in COVID-19 and the European Academy of Neurology Neuro-COVID Registry after anosmia and ageusia were added to the list of symptoms, more precisely from March to September or October 2020.

In this context, our objective is to perform a retrospective analysis of Twitter data to investigate to what extent anosmia and ageusia were reported and associated with COVID-19 in the tweets before their official recognition. As these symptoms are uncommon outside of COVID-19, their unique representation and potential as early indicators of the disease can help differentiate COVID-19 from other respiratory diseases such as the flu. Having detected that anosmia and ageusia were COVID-19 symptoms earlier could have helped diagnose and isolate some patients. Therefore, the findings of this retrospective study contribute to the broader field of infoveillance, providing insights into the utility of Twitter data for early detection and monitoring of symptoms of a new disease.

## Methods

### Tweet Extraction

We considered all the tweets posted between January 1, 2020, and March 31, 2020, which corresponds to the early stages of the pandemic in France. The Detec’t (Kap Code) tool [14] was used to extract all nonretweet tweets in French containing at least one of the keywords related to the sense of smell (eg, anosmia, smell, and olfactive) or taste (eg, ageusia, taste, and gustative) and posted during the first 3 months of 2020. The established list of French keywords and their English translation is given in Table S1 in Multimedia Appendix 1.

An additional data set of tweets posted in early 2019 was also extracted to enable the comparison between 2019 and 2020. The objective of this comparison was to assess if the volume of declarations of anosmia or ageusia with unknown causes in early 2019 was lower than the one in early 2020, which could imply that tweets in early 2020 were related to COVID-19.

### Symptom Detection

Anosmia and ageusia specific data sets were created based on symptom detection in tweets. A fuzzy matching method was applied to the set of smell- or taste-related tweets. More precisely, we reused the dictionary of symptoms based on MedDRA (International Council for Harmonisation of Technical Requirements for Pharmaceuticals for Human Use) and enhanced by colloquial terms, which includes the preferred terms (PTs), the associated low level terms, and manual enrichment [15]. Terms associated with the 2 PTs “anosmia” and “ageusia” were used for detection, based on the method that we previously developed and tuned [15], that is, exact matching for short terms containing less than 6 characters and fuzzy matching for other terms. This method was proven to be effective in improving the detection of symptoms with spelling mistakes or in the plural form. Tweets containing these terms were used to populate the anosmia and ageusia specific data sets.

### Media Coverage of Symptoms

To determine whether there was a temporal gap between the declaration of anosmia and ageusia by patients on the internet and their coverage by media, we compared the coverage of these symptoms in social media and in traditional media. Anosmia- and ageusia-related news articles were extracted using Brandwatch Consumer Research (Brandwatch) tool [16]. Content classified as “News” was counted to describe traditional media coverage over time. Cross correlation [17] between volumes of tweets and news were computed for each symptom to measure the similarity between the series. Dissimilarities would suggest the identification of a temporal gap.

### Tweet Annotation

We considered 2 periods of time to perform random sampling for the tweet annotation: before and after March 10, 2020. The choice of this date was based on the number of cases in France, and the official alert of anosmia and ageusia as COVID-19 symptoms. The first period (January 1-March 10) is an early stage of the epidemic (daily new cases<400, cumulative cases<1500), and the second period (March 10-31) corresponds to the rapid spread of the epidemic, as 1 week after the beginning of the second period, the lockdown started (March 17, 2020), and 3 days later anosmia or ageusia was confirmed as symptoms of COVID-19 by the French health authorities (March 20, 2020). Manual annotation was performed by one of the authors (CF) for a set of randomly selected tweets of the same sample size for period #1 and #2 of 2020 and the corresponding period #1 of 2019, for both symptoms. This resulted in 6 annotated data sets. The goal of this annotation task was to identify mentions of symptoms that could be attributable to COVID-19.

**Figure 1:**
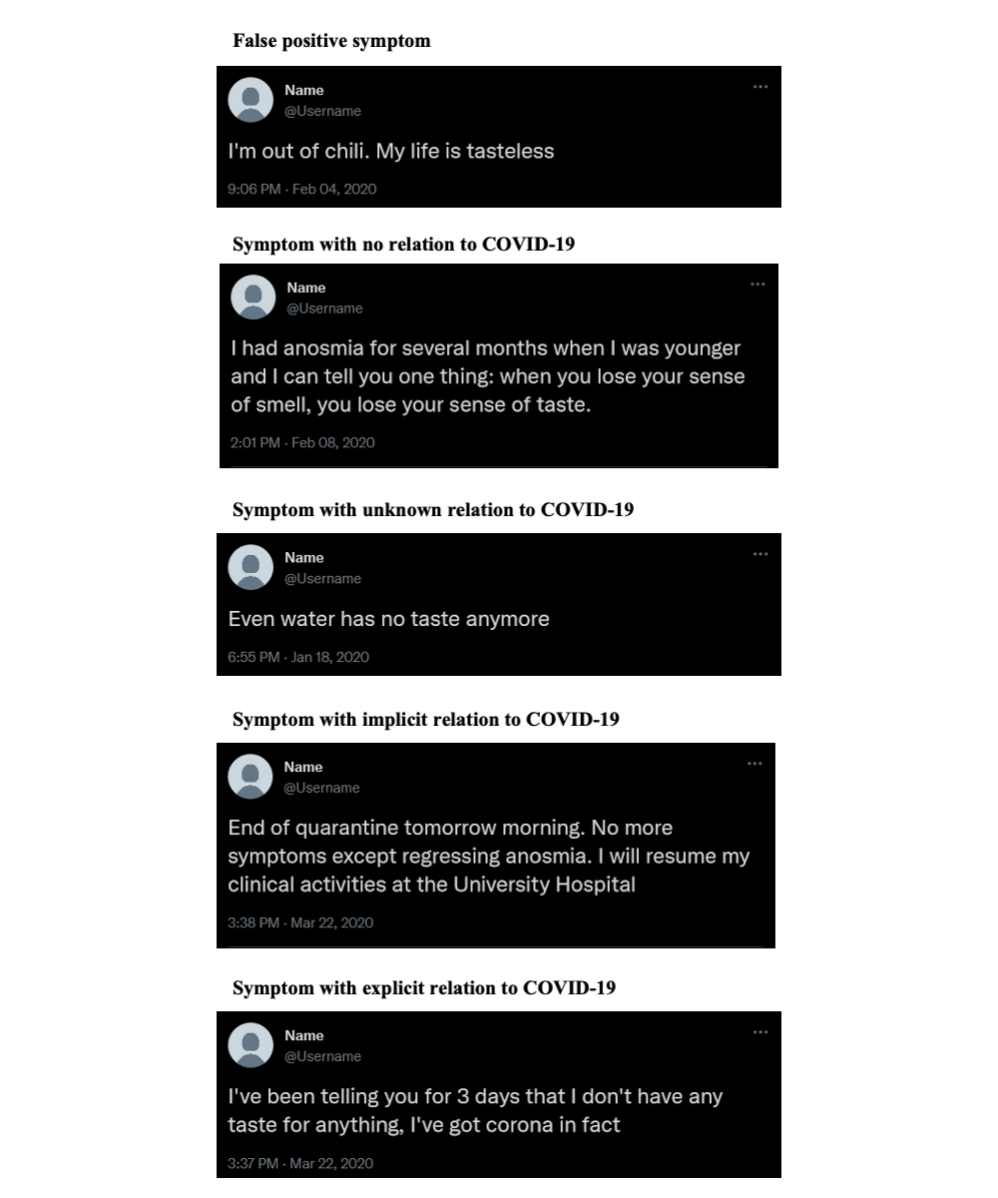
Examples of ageusia- and anosmia-related tweets translated from French to English.

In total, 3 types of annotations were performed. For each detected term, we first annotated if it corresponded to a genuine symptom, that is, reference to a lack of smell or to a lack of taste, or to another meaning. Indeed, a tweet can contain a term from MedDRA associated with the PT “anosmia” and “ageusia” without being a symptom, for example in case of polysemy. For example, terms like “pas de gout” (no taste) may denote ageusia (yes=true positive), but may be also used in other contexts like fashion, thus corresponding to a false positive. Then for each genuine symptom, we further annotated if it was COVID-19–related (yes, no, or unknown), and if, according to the author of the tweet, it was a symptom experienced by an individual (yes, no, or unknown), either the author of the tweet, a relative, or another patient. Regarding the relation to COVID-19, we considered that a tweet was COVID-19–related when the relation was explicitly mentioned by the author or strongly suspected (implicit relation) based on contextual information and the interpretation by the annotator. Figure 1 displays 5 examples of annotated ageusia-anosmia related tweets translated from French.

The fourth example in Figure 1 does not explicitly mention COVID-19 but mentions quarantine which is highly suggestive of an infection by COVID-19. The fifth example expresses ageusia and links it with COVID-19 (“corona”).

### Analysis and Comparison Between Periods and to the Previous Year

Temporal evolution of symptom declarations was analyzed in 2 ways. First, we assessed the temporal evolution of volumes of tweets in each annotation category (symptom yes or no, related to COVID-19 yes, no, or unknown, and experienced yes, no, or unknown). A 3-day cumulative time series were displayed for the 2 periods of 2020 to smooth potential missing data due to the sampling. Second, we focused on the volumes of tweets regarding experienced symptoms with unexplicit causes, and compared them in period #1 of 2020 and 2019. A significant increase in early 2020 compared with early 2019 could suggest that tweets in early 2020 were related to COVID-19 and consequently that cases of COVID-19–related anosmia or ageusia could have been detected earlier in Twitter.

### Ethical Considerations

This retrospective and observational study is not a human subject research as it is based on the secondary analysis of publicly available data for public health research. Therefore, institutional review board approval is not required. Despite that, ethical issues were still considered: usernames were deidentified by removing account identity and other personal information such as email address and tweets cited and discussed were paraphrased (translated from French to English) thus nonreidentifiable.

## Results

### Extracted Tweets With Symptoms of Interest

**Figure 2:**
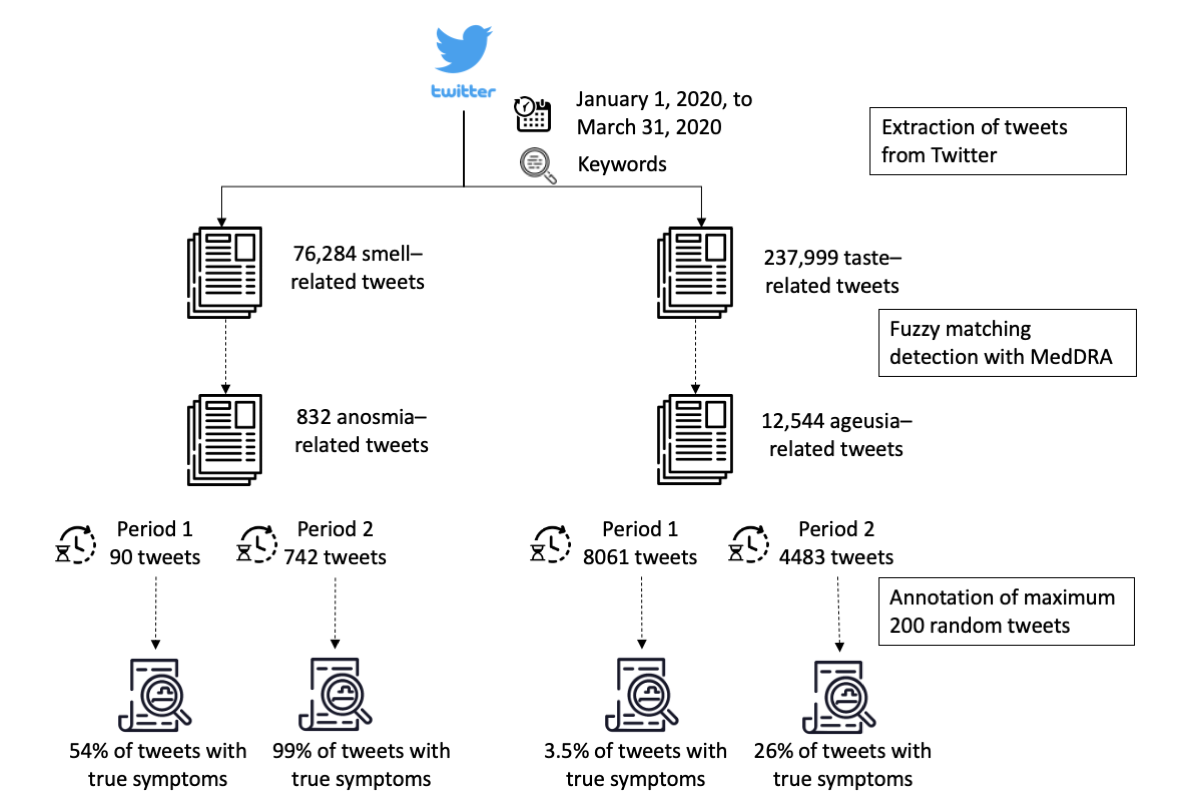
Flowchart of the analysis process.

A set of 307,290 French tweets posted between January 1, 2020, and March 31, 2020, was extracted. Further, 76,284 tweets were obtained with smell-related terms, and 237,999 tweets were obtained with taste-related terms. Symptom fuzzy matching identified 832 tweets containing at least 1 term potentially related to anosmia (90 tweets for period #1 and 742 for period #2) and 12,544 tweets containing at least 1 term potentially related to ageusia (8061 tweets for period #1 and 4483 for period #2). Similarly, 94 tweets potentially related to anosmia and 4721 tweets potentially related to ageusia were extracted for period #1 in 2019. For each period and each symptom of interest, 200 randomly selected tweets were manually annotated. If less than 200 tweets fit the criteria, all the tweets were manually reviewed and annotated, for example, 90 tweets for anosmia in period #1 in 2020. Figure 2 displays a flowchart of the extraction process and the associated volumes of tweets at each step and for each period of 2020.

### Media Coverage

**Figure 3:**
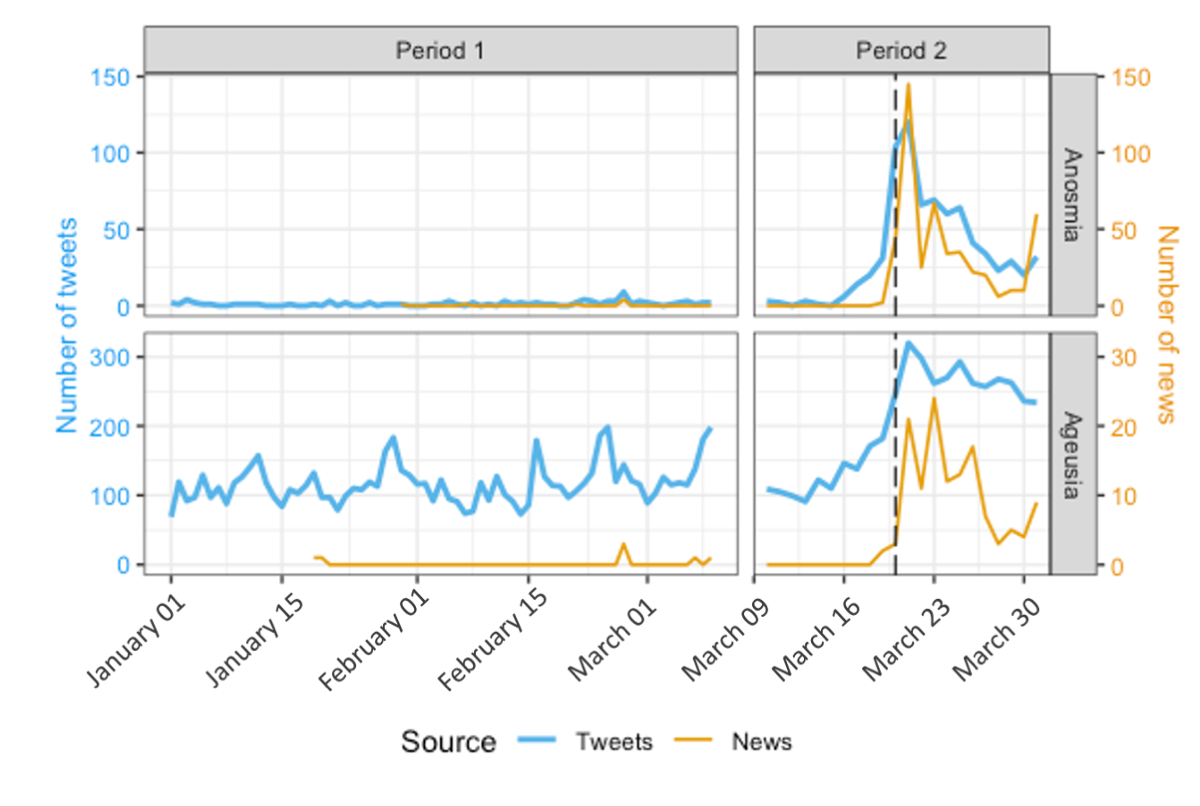
Comparison of the evolution of anosmia- and ageusia-related tweets and news articles between January, 2020, and March 2020.

In total, 549 unique news articles related to anosmia, ageusia, or both were extracted. Only 11 (2%) of them were published in period #1. We compared the temporal evolution of the number of new articles containing anosmia and ageusia to that of tweets (Figure 3). A slight volume peak can be identified for anosmia on the 27th of February, which is Anosmia Awareness Day. A steep increase is observable for both sources on March 20 when these symptoms were officially linked to COVID-19 (denoted as a vertical perforated line). We can see that the increase in the number of publications happens a few days earlier for the tweets than for the news. However, computed cross-correlations show that series are correlated at 87% (anosmia) and 77% (ageusia) without lag, which suggests that there is no significant temporal gap between the declaration of anosmia and ageusia by patients on the internet and their coverage by media. Nevertheless, it shows a strong reactivity of Twitter to events.

### Detailed Analysis of Anosmia-Related Tweets

Regarding anosmia, among the 290 manually annotated anosmia-related tweets, 247 tweets contained genuine anosmia symptoms (49/90 and 198/200 tweets during periods 1 and 2, respectively), 36 were false positives (34/90 and 2/200 tweets during periods 1 and 2, respectively), and 7 tweets from period 1 were too short or unexplicit (eg, presence of irony) to conclude.

Among the 247 tweets containing mentions of the symptom anosmia:

- 165 tweets contained general information or discussions regarding anosmia, without any description of personal experiences (34 in period #1 and 131 in period #2). In total, 95/165 of these tweets were COVID-19–related. The remaining tweets (70/165) addressed topics such as the Anosmia Awareness Day, which is observed on February 27th every year as well as general information regarding anosmia and irony tweets.
- 67 tweets reported on experienced anosmia (15 in period #1 and 52 in period #2). Further, 32/67 tweets were COVID-19-related. Two-thirds of them provided information regarding co-occurring symptoms and about half of them provided the chronology of symptoms. The non–COVID-19–related tweets included mentions of long-term anosmia, and anosmia due to stuffy nose. Interestingly, there were 30 cases of anosmia without any explicit cause (11 in period #1 and 19 in period #2), that might be due to COVID-19 (eg, “I have not been able to smell anything for three days without having a stuffy nose”).
- 15 tweets from period #2 could not be classified.

Regarding the relation to COVID-19, anosmia related to COVID-19 was only found during period #2 (136 tweets). A quarter of these tweets reported on experienced anosmia (32/136).

To summarize the results for anosmia: (1) the performance of the detection method was high, with a precision of 85% (247/290); (2) before March 10, 2020, only 90 tweets containing a term related with anosmia were identified, leading to manually reviewing a total of 290 tweets; (3) among these 290 tweets, a total of 32 tweets (11%) reported experiencing anosmia associated with COVID-19. All these tweets were found during period #2; (4) the World Anosmia Day, on February 27, generated more tweets on anosmia, unrelated to COVID-19; and (5) some qualitative information such as co-occurring symptoms and the temporality of symptoms could be found in a large proportion of tweets from patients with COVID-19.

### Detailed Analysis of Ageusia-Related Tweets

As with anosmia, the first step consisted in solving polysemy issues, that is, differentiating between terms that are related to the symptom ageusia and other polysemic usages of the terms in other contexts (eg, no taste in fashion or music and really insipid food). The initial corpus of tweets with terms potentially related to ageusia was very noisy: among the 400 manually annotated ageusia-related tweets, only 59 tweets (14.8%) contained genuine ageusia symptoms (7 and 52 tweets during periods 1 and 2, respectively), whereas 330 were false positives (186 and 144 tweets during periods 1 and 2, respectively), and 11 tweets were too short or unexplicit (eg, ironic messages) to conclude (7 and 4 during periods 1 and 2, respectively).

Among the 59 tweets containing mentions of the symptom ageusia, (1) 12 tweets contained general information or discussions regarding ageusia, without any description of personal experiences (1 in period #1 and 11 in period #2); (2) 43 tweets reported on experienced loss or absence of taste (6 in period #1 and 37 in period #2). Eighteen tweets out of 43 (41.9%) were COVID-19–related; and (3) 4 tweets could not be classified (all of them after March 10).

Regarding the relation to COVID-19, a large proportion of tweets mentioning the symptom ageusia did not mention the cause (33/59). Ageusia related to COVID-19 was only found during period #2 in 42.4% of the tweets (25/59). Further, 72% of these tweets reported experienced ageusia (18/25), including 7 tweets on its negative psychological impact (eg, “(...) since friday, complete ageusia and anosmia. Life has become really sad, I mean really really sad” [March 24, 2020].

To summarize the results for ageusia: (1) the overall precision of the symptom detection method was very low, with less than 15% (59/400) of the tweets containing genuine ageusia symptoms. This was due to the presence of highly polysemic terms (eg, “goût”); (2) before March 10, there was no post reporting on ageusia experienced by a patient with COVID-19 while after March 10, we found 18 tweets out of 200 (9%) reporting on it; and (3) patients share qualitative information, such as the psychological negative impact of ageusia.

### Time Evolution

In this section, the temporal evolution of declared symptoms and their links to COVID-19 are discussed. We provide results based on the tweets that have been manually reviewed for each symptom and each period. Evolution over time of symptoms declaration, link to COVID-19 and experience of the symptom by the author is studied and plotted.

**Figure 4:**
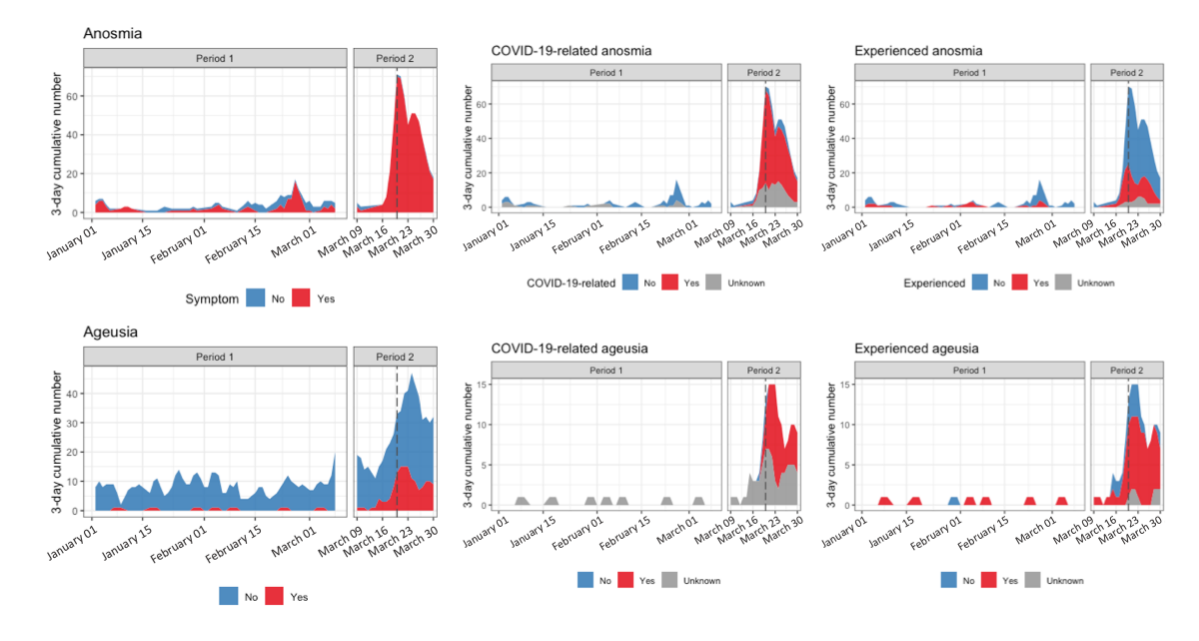
A 3-day cumulative time evolution of manually reviewed anosmia- and ageusia-related tweets. Occurrences of genuine anosmia and ageusia in all the tweets that were manually reviewed (left). True positive symptoms are broken down in proportions of the tweets expressing COVID-19–related symptoms or not (middle) and experience of symptoms (right).

The 3-day rolling total number of tweets in each annotated category was calculated for each symptom. The evolution of proportion for all annotation groups is shown in Figure 4. The proportion of tweets related to the symptom ageusia among ageusia-related tweets (Figure 4, left) increased from the beginning to the end of the second period but remained low (from 3.5%, 7/200 before March 10 to 26%, 52/200 after, see Figure 2). As for anosmia, the proportion of tweets with genuine symptoms was 54% (49/90) before March 10 contrasting with 99% (198/200) after (Figure 2 and Figure 4, left).

The number of tweets relating experienced symptoms during period #1 was very low for both ageusia and anosmia (Figure 4, right). Over period #2, despite a global increase in the number of tweets, the proportion of tweets reporting on experienced symptoms did not drastically evolve, and remained very low for anosmia and important for ageusia. Regarding COVID-19–related mentions of symptoms (Figure 4, middle), tweets associated with COVID-19 were identified only from March 17 for anosmia and March 20 for ageusia. The proportion of tweets relating COVID-19 symptoms among tweets relating genuine symptoms reached 62% (21/34) during the last 10 days of period #2 for ageusia, contrasting with 22% (4/18) during the beginning of period #2. For anosmia, the proportion remained high and quite constant over the whole period #2, around 69% (136/198). These results show the important impact on Twitter of the media coverage of these symptoms, which confirms the high reactivity of Twitter to news, and the fact that Twitter can be a source of interest to find qualitative information regarding an epidemic.

As these symptoms were confirmed as related to COVID-19 by Health authorities on Friday, March 20th, we performed a deeper analysis to assess if experienced COVID-19 related anosmia or ageusia (experienced=yes, COVID-19–related=yes) were detected on social media before this date, at the very beginning of period #2. The evolution of the 3-day cumulative number of tweets related to symptoms (all, COVID-19–related, experienced) showed a peak on March 20 and March 23 for anosmia and ageusia, respectively, but some tweets related to COVID-19 could be found a few days earlier.

Regarding anosmia, 6 tweets were found before March 20 (from March 17 to March 19) mentioning (explicitly or implicitly) experienced COVID-19 symptoms including anosmia. Further, 5 out of 6 tweets were written by health professionals about their patients (eg, “I started to get it the third time, generally I see one patient for anosmia per month, this time it is 3 in 48 hours” [March 17, 2020]). Further, 1 tweet was written by a web user who had experienced COVID-19 with anosmia (“I share this diagnosis because it is exactly what I have had for 2 days (...) some of my relative also have the same symptoms #anosmia # Covid” [March 19, 2020]). Some tweets posted before mentioned experienced anosmia but the relation to COVID-19 cannot be verified (eg, “has someone already had anosmia? #anosmia #help” [February 14, 2020], “I wear perfume but I don’t smell it, I brush my teeth but I don’t even taste the tooth path... It is really hard for me because I usually pay a lot of attention to smell” [January 23, 2020]).

Regarding ageusia, no tweet could be found before March 20 mentioning experienced COVID-19 symptoms including ageusia. Experienced ageusia with unexplicit cause could be found earlier, with unknown (eg, “even my favorite meals don’t taste anything anymore” [January 8, 2020], “even water doesn’t taste anything anymore” [January 18, 2020]) or high probability (eg, “Yes... I smelt something for the first time after 10 days, I still don’t taste anything anymore” [January 19, 2020]) to be associated with COVID-19.

### Comparison With 2019

To assess whether a signal about anosmia and ageusia could have been detected from Twitter, we compared the number of tweets mentioning experienced ageusia or anosmia with unexplicit causes (experienced=yes, COVID-19–related=unknown) posted during the same periods in 2019 and 2020. Using the same methodology as with 2020, we extracted 105 and 4701 tweets for anosmia and ageusia from January 1, 2019, to March 9, 2019, respectively. All anosmia-related tweets and 200 randomly sampled ageusia-related tweets were manually annotated.

Regarding anosmia, 75 tweets (respectively 49) mentioning the anosmia symptom were identified in 2019 (respectively 2020). Among them, 15 tweets (respectively 11) mentioning experienced anosmia without any cause were found.

Regarding ageusia, 8 tweets (respectively 7) mentioning the ageusia symptom were identified in 2019 (respectively 2020), and among them 7 tweets (respectively 6) mentioning experienced ageusia without any cause were found.

We could not identify any notable quantitative or qualitative difference between 2019 and 2020 that could have been early signals for COVID-19–related anosmia and ageusia in 2020.

## Discussion

### Principal Results

In this paper, our objective was to assess if the association between anosmia or ageusia and COVID-19 could have been detected earlier by mining social media, that is, before the relation was identified and published in journals. We tested such a hypothesis on a set of French tweets posted between January, 2020, and March 2020. We focused on these two symptoms for three reasons: (1) they were associated with COVID-19 later than other symptoms, these terms were even little known by the population before this pandemic, (2) they were present in a large proportion of the population with moderate symptoms in Europe but less in Asia earlier, and (3) they are quite uncommon outside of COVID-19, so their potential as early indicators of the disease could have helped differentiate COVID-19 from other diseases. Regarding the comparison with news articles, computed cross-correlations showed a strong correlation between the temporal evolution of the number of new articles containing anosmia and ageusia to that of tweets without lag, which suggests that there was no significant temporal gap between the declaration of anosmia and ageusia by patients on the internet and their coverage by media. Nevertheless, these results show a strong reactivity of Twitter to news. To obtain more insights, given the significant noise in symptom extraction, we performed a manual review of the anosmia- and ageusia-related tweets. We showed that only 6 tweets associating anosmia to COVID-19 could be identified among our set of manually annotated tweets before the news coverage, and these tweets were posted only 3 days before. Further, 5 out of 6 of these early warning messages (83%) were written by health professionals. No association could be found earlier. These results tend to demonstrate that Twitter could not have helped to identify the relation between COVID-19 and anosmia or ageusia during the early stage of the pandemic. Eventually, to assess if anosmia- and ageusia-related tweets with unexplicit causes from early 2020 could have been due to COVID-19, we compared their volume to the number of tweets mentioning experienced ageusia or anosmia with unexplicit causes from the same period from 2019. However, we could not identify any notable quantitative or qualitative difference between 2019 and 2020.

Most of the tweets associating anosmia or ageusia and COVID-19 were posted later, that is, after March 20th. These tweets contained qualitative information, such as psychological impact for ageusia and chronology of associated symptoms for anosmia, which could be of interest for further analyses. For example, it could be interesting to compare such insights with information from more traditional sources, especially as, according to Sarker et al [18], these symptoms were not reported in clinical studies at this time. We think the reactivity of Twitter can make it a useful tool to provide qualitative information that could complement what is already known in case of any health alert.

### Comparison With Prior Work

A PubMed search using “social media” and “covid” as keywords retrieved 6764 publications, while adding the keyword “symptoms” restricted the results to 750 publications. Indeed, many prior studies explored the topic and sentiment expressed in social media (eg, [3,4,19,20]) misinformation and fake news spread in social media during COVID-19 (eg, [21-23]), and mental health (eg, [24,25]). As for symptoms, some efforts were made to improve the extraction of COVID-19–related symptoms from social media tweets for further investigation without a specific infoveillance objective (eg, [15,26]). Shen et al [27] performed an observational infoveillance study with a focus on the early stage of COVID-19 outbreak in China (before March 31, 2020) by assessing the relationship between reports of symptoms in Chinese social media platform Weibo and the daily confirmed infected cases. Ding et al [28] performed an infodemiological study to assess how discussion of symptoms changed over time and to identify correlations between frequency of the top 5 commonly mentioned symptoms in post and daily COVID-19 statistics (new cases and new deaths) in the United States. However, anosmia and ageusia were not mentioned in these 2 studies. Indeed, Shen et al [27] established a broad list of symptoms that did not include anosmia and ageusia probably due to the low prevalence of these symptoms observed in China during the first 3 months of 2020 [8], and Ding et al [28] focused on the top 5 commonly and most mentioned symptoms. Regarding anosmia and ageusia, Sarker et al [18] found that these 2 symptoms were frequently reported in English tweets, but not in clinical studies, and consequently that COVID-19 symptoms identified from Twitter may complement those identified in clinical settings. However, they performed their analysis in May 2020 and did not analyze the temporal characteristics of the distributions on Twitter, so it is unclear if reported anosmia and ageusia symptoms were found before the media coverage. Sudre et al [29] measured the association between different symptoms and COVID-19, based on data collected through 6 surveillance platforms and demonstrated that anosmia or ageusia was the strongest, most consistent symptom of COVID-19. However, they used the data collected between April, 2020, and June 2020, which is later after the official announcement. In this study, we conducted a comprehensive analysis of French tweets between January, 2020, and March 2020, focusing on the early detection and association of anosmia and ageusia with COVID-19. The temporal evolution of declared symptoms and their association with COVID-19 was assessed, allowing us to gain insights into the temporal trends and patterns of anosmia and ageusia discussions on Twitter. The comparison with media coverage and the comparison with the anosmia and ageusia related tweets in 2019 strengthened our conclusion.

### Limitations

**Figure 5:**
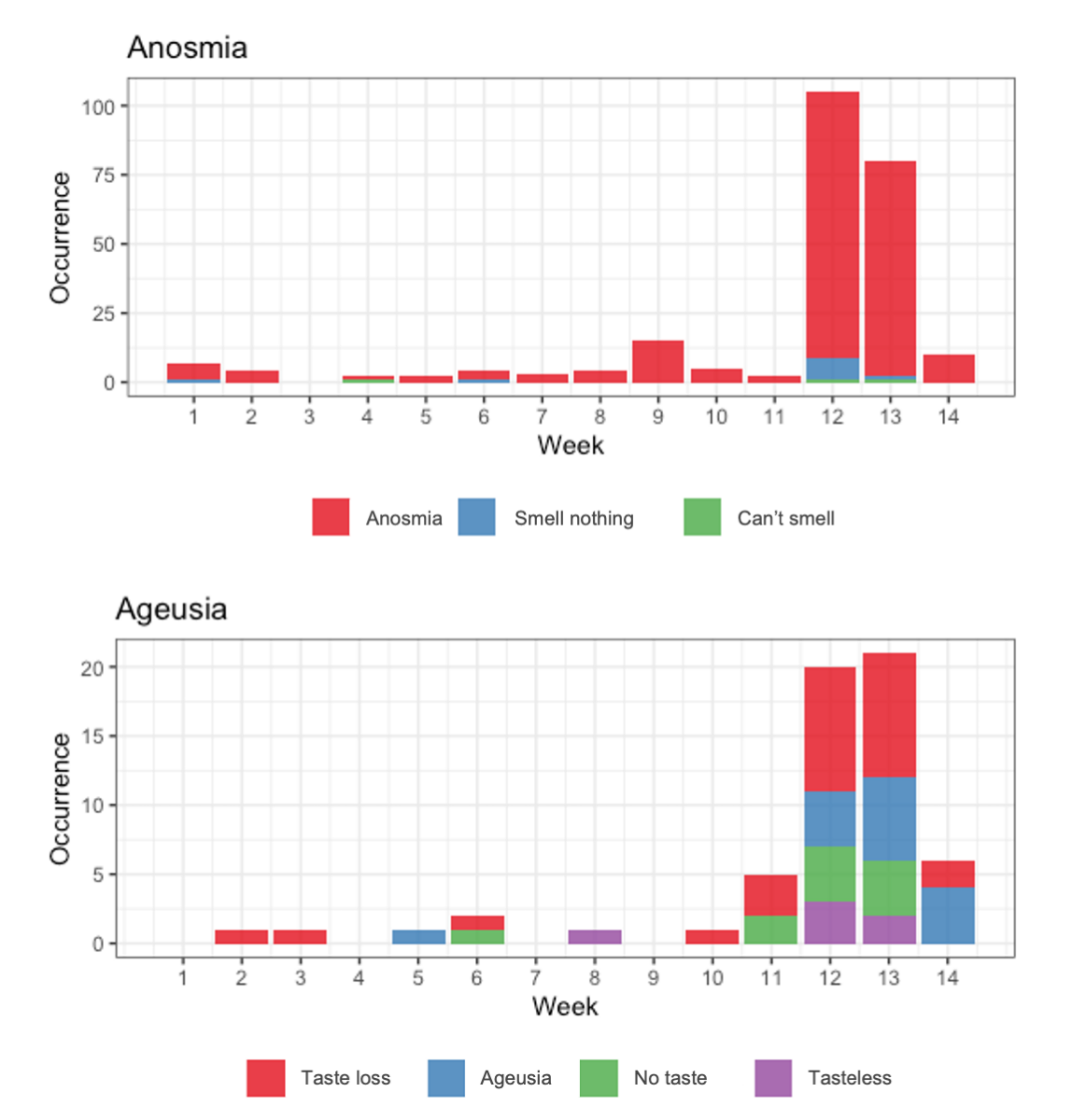
Anosmia- and ageusia-related terms for symptom detection.

The exact terms “ageusia” and “anosmia” were quite unknown by the lay public before the overmediatization of these symptoms. Figure 5 shows the different words used to express COVID-19–related symptoms over our analysis period. The word “agueusie” per se is rarely used to denote this symptom before March 20 (week 12). After this date, its use takes up a growing proportion of the vocabulary used. This suggests an effect of mediatization on how patients express their symptoms, which shows a positive evolution in patients' health literacy through media outreach, also thanks to Twitter reactivity. In contrast, the medical term “anosmie” is the dominant term to express loss of sense of smell over the whole period. This can be explained by the tweets about the Anosmia Awareness Day on February 27, an international event that brings attention to the patients without the sense of smell, whatever the cause.

The absence of use of precise medical terms in the tweets made it more difficult to extract these symptoms, especially before March 20, 2020. We considered synonymous terms for symptom detection, but these synonyms are much more polysemous: for example, “pas de goût” (no taste) can have different meanings, including taste, flavor, liking, fondness, and preference; consequently, on the evaluation subset, only 59 out of 400 (14.7%) ageusia-related tweets were actually dealing with the symptom ageusia.

### Conclusions

Based on our analysis of French tweets, associations between COVID-19 and anosmia or ageusia by web users could have been found on Twitter only a few days before the official announcement but not during the early stage of the pandemic. The comparison between early 2020 and early 2019 showed no difference regarding the volumes of tweets, and no significant temporal gap between the declaration of anosmia and ageusia by patients on the internet and their coverage by media could be found.

Although we could not find early signals linking anosmia or ageusia with COVID-19 on Twitter, these tweets can potentially provide valuable information. First, we have demonstrated that Twitter was highly reactive to the news, with a significant increase after March 20. Second, our analysis showed that tweets contained qualitative information, such as psychological impact for ageusia and chronology of associated symptoms for anosmia. We think the reactivity of Twitter can make it a useful tool to provide qualitative information that could complement what is already known in case of any health alert.

## Appendix

Multimedia Appendix 1 List of anosmia and ageusia related keywords for the extraction.

## Abbreviations

PT: preferred term

## Data Availability

The data sets generated and analyzed during this study are not publicly accessible in accordance with European Union General Data Protection Regulation requirements. However, some anonymized examples can be obtained from the corresponding author upon reasonable request.
